# Efficacy, safety and mechanistic insights of pentoxifylline in major depressive disorder: a systematic review and meta-analysis of randomized controlled trials

**DOI:** 10.1007/s00210-025-03845-1

**Published:** 2025-02-22

**Authors:** Omar Kassar, NourAllah Farag, Abdullah Selim, Lamees Taman, Menna Alaa, Ahmed Elshahat, Moaz Elsayed Abouelmagd

**Affiliations:** 1 Faculty of Medicine, Alexandria University, Alexandria, Egypt; 2 Faculty of Medicine, Suez University, Suez, Egypt; 3 Faculty of Medicine, Tanta University, Tanta, Egypt; 4 Medical Research Group of Egypt, Neida Academy, Arlington, MA USA; 5 Shebin Elkom Hospital for Mental Health & Addiction Therapy, Menoufia, Egypt; 6 Faculty of Medicine, Al-Azhar University, Cairo, Egypt; 7 Faculty of Medicine, Cairo University, Cairo, Egypt

**Keywords:** Depression, Inflammation, MDD, Meta-analysis, Pentoxifylline, Serotonin

## Abstract

Novel treatments that act beyond the conventionally targeted monoamine system are urgently needed to provide more effective relief for patients with major depressive disorder. Pentoxifylline (PTX) is a phosphodiesterase inhibitor with potent anti-inflammatory and antioxidant effects, with additional pleiotropic effects. This is the first systematic review and meta-analysis to examine the role of PTX in major depressive disorder. A comprehensive search of electronic databases, including PubMed, Scopus, Cochrane, and Web of Science, was performed in October 2024. We included only randomized controlled trials (RCTs), and their data were extracted and analyzed using Reman 5.4 software. The primary outcome was the change in Hamilton Depression Rating Scale (HAM-D). Four RCTs with 318 patients were included in the study. PTX showed a statistically significant improvement in HAM-D scores at the primary endpoint compared to the placebo (MD = –3.84, 95% CI [–4.87 to –2.81], *P* < 0.00001). Moreover, PTX showed a statistically significant increase in serotonin and BDNF levels (MD = 20.76 ng/mL, 95% CI [5.49 to 36.04], *P* = 0.008; and MD = 10.83 ng/mL, 95% CI [-0.22 to 21.88], *P* = 0.05, respectively) and a statistically significant decrease in TNF-α and IL-6 levels (MD = –3.24 pg/mL, 95% CI [–4.12 to –2.36], *P* < 0.00001; and MD = –2.64 pig/mL, 95% CI [–3.79 to –1.48], *P* < 0.00001, respectively). There was no statistically significant difference between the PTX and placebo in any of the reported side effects. The study findings suggest that PTX may be effective and safe as an adjuvant antidepressant agent in patients with MDD, demonstrating a significant reduction in HAM-D scores. The results of this study need to be interpreted with caution considering several limitations.

**Supplementary Information:**

The online version contains supplementary material available at 10.1007/s00210-025-03845-1.

## Introduction

Major depressive disorder (MDD) is a serious and prevalent psychiatric disorder that affects the lives of millions of people around the world. MDD patients suffer from mood abnormalities, sleep and appetite disturbance, and feelings of worthlessness and guilt, which affect the quality of life and the ability to work (Fava and Kendler 2000). Additionally, it has been linked to increased risk of several chronic diseases, such as diabetes and cardiovascular diseases (Chapman et al. 2005; Gutiérrez‑Rojasetal., 2020).

MDD is a multifactorial disease with several risk factors including genetic, biological, psychological, hormonal, and environmental factors. These factors contribute to several key pathways implicated in the pathophysiology of MDD, such as vascular dysfunction, immune system dysregulation, oxidative stress, and changes in synaptic plasticity. Additionally, the biogenic monoamine theory suggests that MDD is associated with abnormalities in neurotransmitters like serotonin, which play critical roles in regulating physiological functions and mood stability (Bains and Abdijadid 2024; Drevets et al. 2007; Jans et al. 2007).

Pentoxifylline (PTX) is a methylated xanthine derivative that exerts biologic effects through nonspecific phosphodiesterase (PDE) inhibition (Aviado and Porter 1984). It is FDA-approved for the treatment of intermittent claudication (Jacoby and Mohler 2004). PTX is a pleiotropic agent with the potential to affect several pathways involved in the dysregulated mechanisms of MDD.

Currently, there is a conflict around the efficacy of PTX in psychiatric disorders. Multiple randomized controlled trials (RCTs) found that PTX has a promising effect as adjuvant therapy in the management of depression (El‑Haggar et al. 2018; Farajollahi-Moghadam et al. 2021). However, Mohammad et al. 2024 found no benefit of PTX in treating bipolar patients with resistant depression except for patients with high inflammatory markers (Mohammad et al. 2024).A systematic review conducted in 2021 found that PTX is beneficial as adjuvant therapy in improving mood and decreasing depressive symptoms, but this study was limited by only one clinical trial (Siegel et al. 2021), so this is the first meta-analysis to investigate the efficacy and safety of PTX in patients with a primary diagnosis of MDD.

## Methods

We followed the Preferred Reporting Items for Systematic Reviews and Meta‐Analysis (PRISMA statement) (Page et al. 2021) guidelines when reporting this study. This manuscript was conducted in adherence to the Cochrane Handbook of Systematic Reviews of Interventions (Cumpston et al. 2019). This study was prospectively registered on PROSPERO (CRD42024603653).

### Search strategy and data sources

We searched PubMed, Web of Science, Cochrane, and Scopus for relevant studies in October 2024. For a sensitive search strategy, we used the MeSH keywords “Pentoxifylline” in combination with “Depression.” The detailed search terms used for each database are illustrated in Supplementary Table 1.

### Selection criteria

Studies meeting the following inclusion criteria were included in the systematic review: (1) Population: adult patients diagnosed with Major Depressive Disorder (MDD), (2) Intervention: pentoxifylline with or without selective serotonin reuptake inhibitors (SSRIs), (3) Comparator: placebo with or without SSRIs, (4) Outcome: studies reporting at least the Hamilton Depression Rating Scale (HAM-D), and (5) Study Design: randomized controlled trials.

We excluded articles if they met any of the following criteria: (1) Patients with comorbid psychiatric disorders or neurological conditions (2) Case reports, single arm studies, case series, observational studies, conference abstracts, or theses, (3) Animal or in vitro studies, (4) Reviews, book chapters, letters, or studies with overlapping datasets, and (5) Studies published in non-English languages.

### Screening and study selection process

Three independent authors (AS, LT, MA) used Rayyan (Ouzzani et al. 2016) for semi-automated screening of the literature search results. Studies were screened in two phases; we screened the titles/abstracts for potential clinical studies in the first phase. In the second phase, we retrieved the full-text articles of the selected abstracts for further eligibility screening.

### Data extraction

For all included studies, data were extracted into an online data extraction sheet by two independent authors (AM and OK), and the extracted data was then compared to confirm accuracy. The extracted data was primarily divided into three domains: (1) study characteristics, (2) characteristics of the study populations, and (3) study outcomes.

### Risk of bias assessment

Two independent authors (LT and NF) assessed the risk of bias using the Online Cochrane Collaboration's Risk of Bias 2 (ROB-2) tool, which evaluates five domains: D1 (bias arising from the randomization process), D2 (bias due to deviations from the intended intervention), D3 (bias due to missing outcome data), D4 (bias in the measurement of the outcome), and D5 (bias in the selection process of reported outcomes). Each study was independently rated as having a low, unclear, or high risk of bias.

### Measures of treatment effectiveness

The primary outcome measures are the Hamilton Depression Rating Scale (HAM-D), response rate (≥ 50% reduction in the HAM-D score), and remission rate (HAM-D score ≤ 7). Other secondary outcomes included:

1. The change in serum levels of the following biological markers: serotonin, brain-derived neurotrophic factor (BDNF), tumor necrosis factor-alpha (TNF-α), interleukin-6 (IL-6), interleukin-10 (IL-10), interleukin-1 beta (IL-1β), 8-hydroxy-2'-deoxyguanosine (8-OHdG), and C-reactive protein (CRP).
2. Adverse events and side effects.
3. Discontinuation rates for any reason.

### Evidence synthesis

We analyzed data using Reman software, version 5.4. For continuous data, the mean difference (MD) between the two groups, along with its standard deviation (SD) and total number of patients, were pooled using the inverse variance method with a random effects model. For dichotomous data, the frequency of events and the total number of patients were pooled as risk ratios (RR) between the two groups, also using the inverse variance method with a random effects model. Heterogeneity was assessed using the chi-square test and I^2^ statistic; a p-value from the chi-square test < 0.1 and I^2^ > 50% were considered indicative of significant heterogeneity. We conducted a subgroup analysis based on whether PTX was taken alone or as an adjunct to SSRIs. We conducted a leave-one -out sensitivity analysis to check if the overall effect was driven by a single study. In studies reporting data with multiple time points, we selected the most consistent point and the endpoint.

### Publication bias

In agreement with Egger et al. (Egger et al. 1997), it was not applicable to examine potential publication bias in our review via Egger's test for the funnel plot asymmetry, when the number of the included studies was fewer than ten studies.

## Results

### Literature search results

**Fig. 1:**
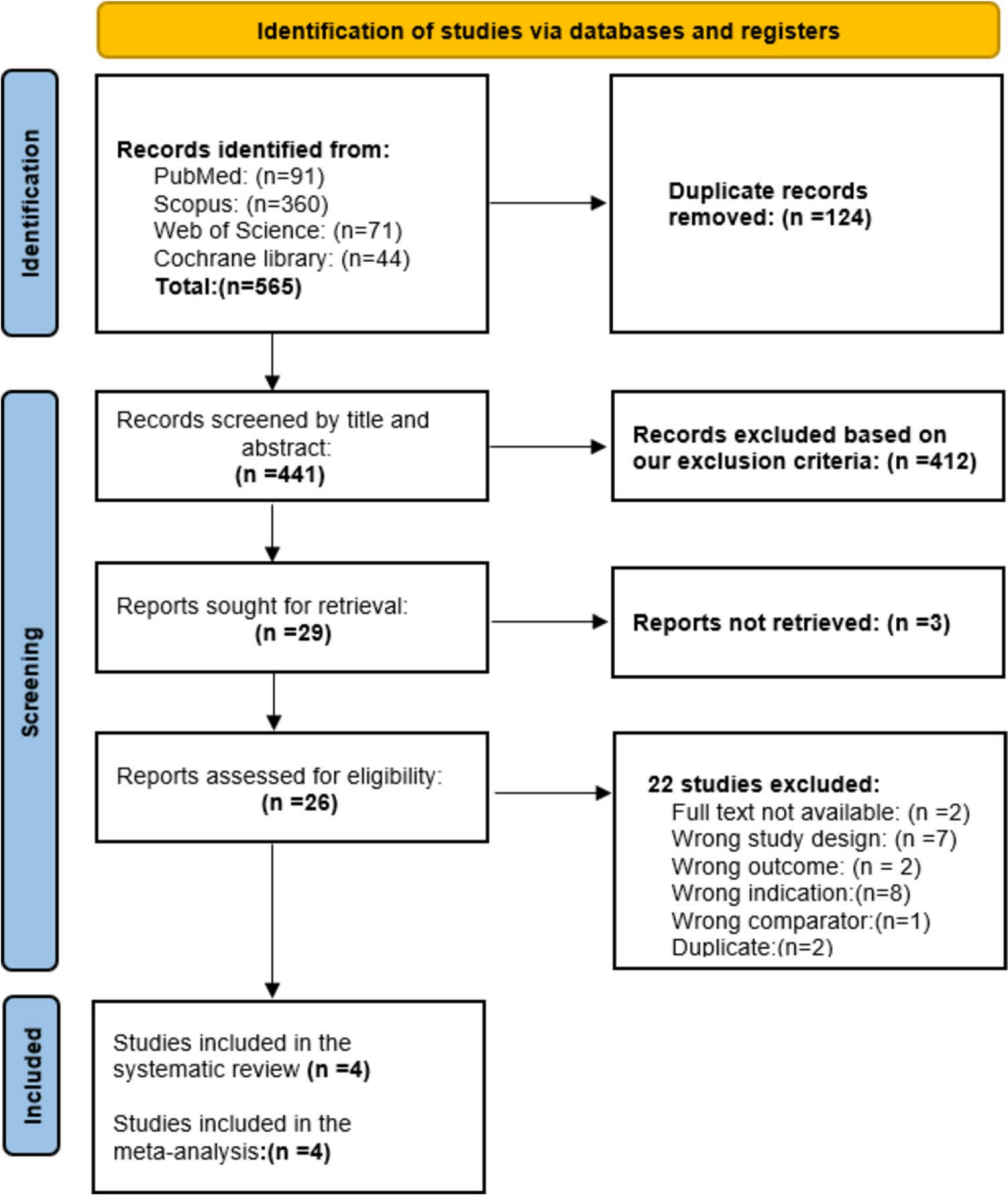
The PRISMA flow diagram for the included studies

We obtained 565 studies from the search, and Rayyan identified 124 duplicates. After excluding irrelevant articles, 29 articles were eligible for full-text screening. Of these, 4 RCTs were included in this systematic review and meta-analysis. The PRISMA flow diagram of the study selection process is shown in (Fig. 1).

### Characteristics of the included studies

All studies were RCTs where the PTX dose was 400 mg twice daily, except for one study (Farajollahi‑Moghadam et al. 2021), in which patients received 400 mg three times daily. All patients in the studies took PTX in combination with SSRIs, except for one study (Yasrebi et al. 2021), which received PTX as monotherapy. Two studies were conducted in Iran (Merza Mohammad et al. 2024; Yasrebi et al. 2021), one in Egypt (El‑Haggar et al. 2018), and one in Iraq (Farajollahi‑Moghadam et al. 2021). Baseline characteristics and a summary of included studies are shown in Tables 1 and 2.

**Table 1:** Summary of the included studies

Study ID	Location	Year	Study design	Population	Sample Size	Intervention	PTX Dose	Comparator	Treatment duration	Evaluation time	Outcome measures	Key findings
El-Haggar et al 2018	Egypt	2015–2017	RCT	Moderate MDD patients	80	PTX plus Escitalopram 20 mg/day	400 mg BID	Placebo plus Escitalopram 20 mg/day	12 weeks	baseline,4,8,12 weeks	HAM-D score / TNF-α, IL-6, IL-10 and levels of BDNF and serotonin	The findings of this study suggest that PTX could be a promising adjunct to antidepressants in the treatment of MDD patients
Yasrebi et al 2021	Iran	2020	RCT	Mild to moderate MDD Patients with CAD	70	PTX	400 mg BID	Placebo	6 weeks	baseline,2,4,6 weeks	HAM-D score	Patients receiving pentoxifylline had greater improvement in HAM-D scores from baseline, Rate of remission, treatment response, and adverse effects did not differ between the two groups
Farajollahi-Moghadam et al 2021	Iran	2019- 2020	RCT	Severe MDD patients	68	PTX plus sertraline 100 mg/day	400 mg TID	Placebo plus sertraline 100 mg/day	6 weeks	baseline,2,4,6 weeks	HAM-D score	The findings support the efficacy and safety of pentoxifylline combination therapy in depression patients with MDD
TAM Mohammad et al 2024	Iraq	2021–2022	RCT	Severe MDD patients	100	PTX plus citalopram 20 mg/day	400 mg BID	Placebo plus citalopram 20 mg/day	12 weeks	baseline, 2,4,6,8,10,12 weeks	HAM-D score and as secondary outcome measures, serum concentrations of IL-6, IL-10, IL-1-β, CRP, TNF-α, BDNF, and serotonin	This study suggests that the PTX add-on, a well-tolerated, promising agent for treating MDD patients

Abbreviations: *PTX,* pentoxifylline; *RCT,* randomized controlled trials; *MMD,* major depressive disorder; *BID,* twice daily, *IL,* interleukin; *BDNF,* brain-derived neurotrophic factor; *CAD,* coronary artery disease; *HAM-D,* Hamilton Depression Rating Scale; *TID,* triple daily; *TNF,* tumor necrosis factor; *CRP,* C-reactive protein

**Table 2:** Baseline characteristics of the included studies

Study ID	Group Name	Age (SD)	Male(percent)	Body weight (SD)	BMI (SD)	Smoking	Marital status: single	HAM-D (SD)	No. of previous MDD Episodes
El-Haggar 2018	PTX	32.73 (8.38)	20(50%)	69.9 (7.34)	24.08 (1.58)	30 (75%)	25 (62.5%)	19.85 (1.39)	7
	Placebo	33.08 (7.59)	19(48.7%)	69.87 (6.42)	24.39 (1.9)	31 (77.5%)	26 (65%)	19.38 (1.25)	6
Yasrebi 2021	PTX	53.8 (3.3)	18 (51.4%)	N/A	N/A	N/A	N/A	15.97 (0.93)	N/A
	Placebo	54.9 (2.8)	15 (42.9%)	N/A	N/A	N/A	N/A	15.72 (1.14)	N/A
Farajollahi-Moghadam 2021	PTX	31.92 (8.46)	16 (57.1%)	68.28 (10.77)	N/A	N/A	N/A	26.07 (3.45)	N/A
	Placebo	34.39 (7.28)	15 (53.6%)	67.71 (10.15)	N/A	N/A	N/A	25.82 (3.00)	N/A
TAM Mohammad 2024	PTX	32.47 (8.31)	27 (54%)	N/A	26.29 (4)	29 (58%)	9 (18%)	27.6 (9)	6.8
	Placebo	30.7 (7.12)	27 (54%)	N/A	27.56 (3.1)	37 (74%)	12 (24%)	26.1 (1.9)	7.1

Abbreviations: All qualitative variables are represented as number (percentage), while quantitative variables are shown in mean (standard deviation). Note: *HAMD*, Hamilton Depression Rating scale; *MDD*, Major Depressive Disorder; *BMI*, Body mass index; *PTX*, Pentoxifylline; *N/A*, not available

### Quality assessment according to risk of bias

**Fig. 2:**
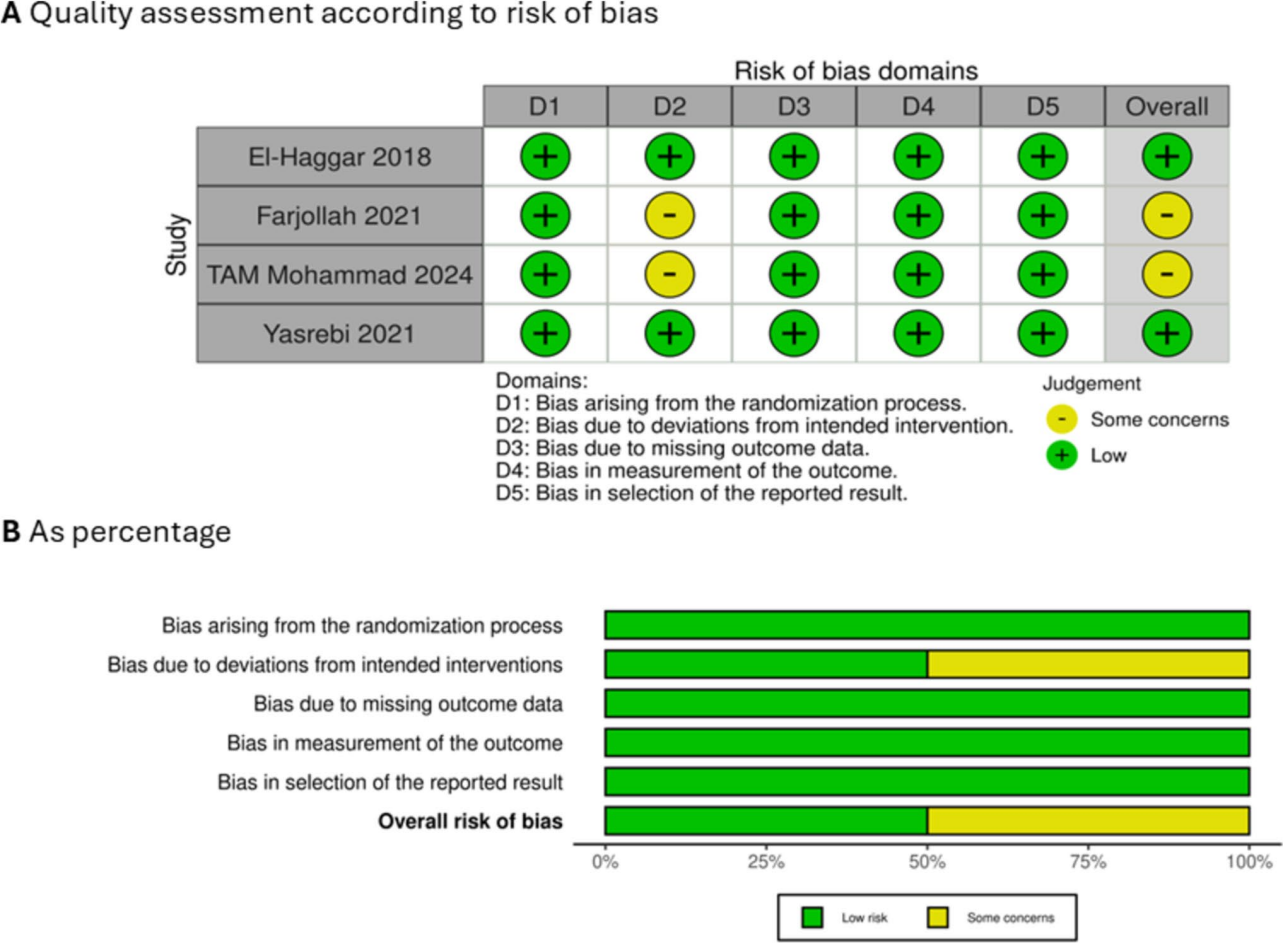
Quality assessments. **A** According to risk of bias for each study. **B** According to risk of bias as percentage

A summary and graph of the risk of bias in our included studies are shown in (Fig. 2). Results of the risk of bias assessment showed that the included studies’ quality varies between low risk and some concerns. Some concerns mainly in domain 2 (deviation from intended interventions) due to inappropriate analysis used to estimate the effect of intervention.

### Primary outcome (HAM-D)

**Fig. 3:**
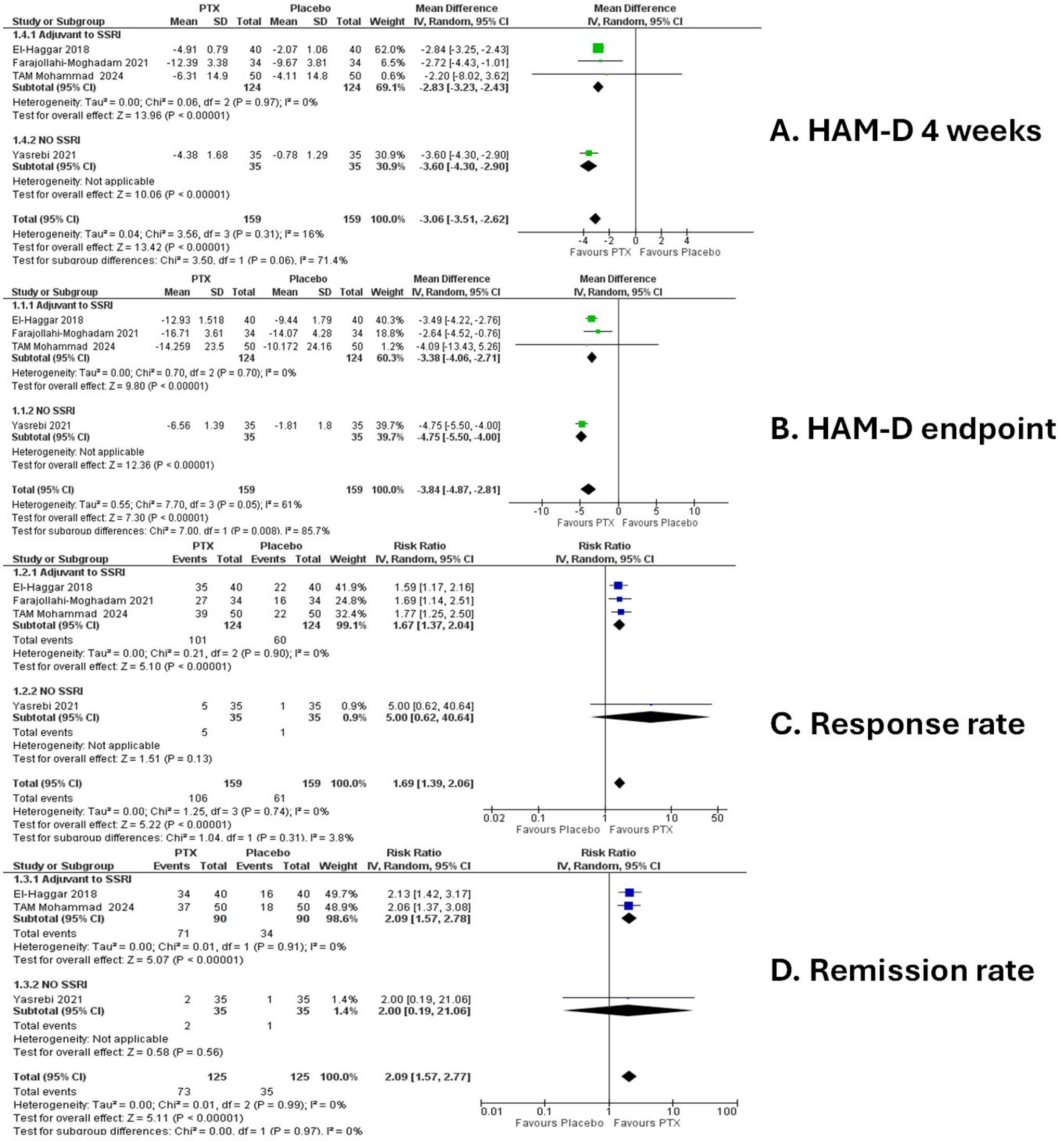
Forest plot of **A** HAM-D scores at 4 weeks, **B** HAM-D scores at primary endpoint, **C** Response rate, **D** Remission rate. IV: Inverse-variance, CI: Confidence interval, PTX: pentoxifylline

Four studies reported the HAM-D score at four weeks. The overall effect statistically favoured PTX over placebo (MD = –3.06, 95% CI [–3.51 to –2.62], *P* < 0.00001). The pooled studies were homogeneous (Chi-square *P* = 0.31, I^2^ = 16%) (Fig. 3A).

This is further confirmed by the analysis of HAM-D score at the post-treatment assessment, where the overall effect statistically favoured PTX over placebo (MD = –3.84, 95% CI [–4.87 to –2.81], *P* < 0.00001). The pooled studies were heterogeneous (Chi-square *P* = 0.05, I^2^ = 61%), as shown in the forest plot (Fig. 3B).

As for the response rate, the overall effect of four studies statistically favoured PTX over placebo (RR = 1.69, 95% CI [1.39, 2.06], *P* = 0.00001). The pooled studies were homogeneous (Chi-square *P* = 0.74, I^2^ = 0%), as shown in the forest plot (Fig. 3C).

Three studies reported the remission rate, the overall effect statistically favoured PTX over placebo (RR = 2.09, 95% CI [1.57, 2.77], *P* = 0.00001). The pooled studies were homogeneous (Chi-square *P* = 0.99, I^2^ = 0%), as shown in the forest plot (Fig. 3D).

We conducted leave-one-out sensitivity analysis by excluding one study in each scenario to check the robustness of our findings. The results statistically favoured ondansetron over placebo in each scenario.

### Biological markers

**Fig. 4:**
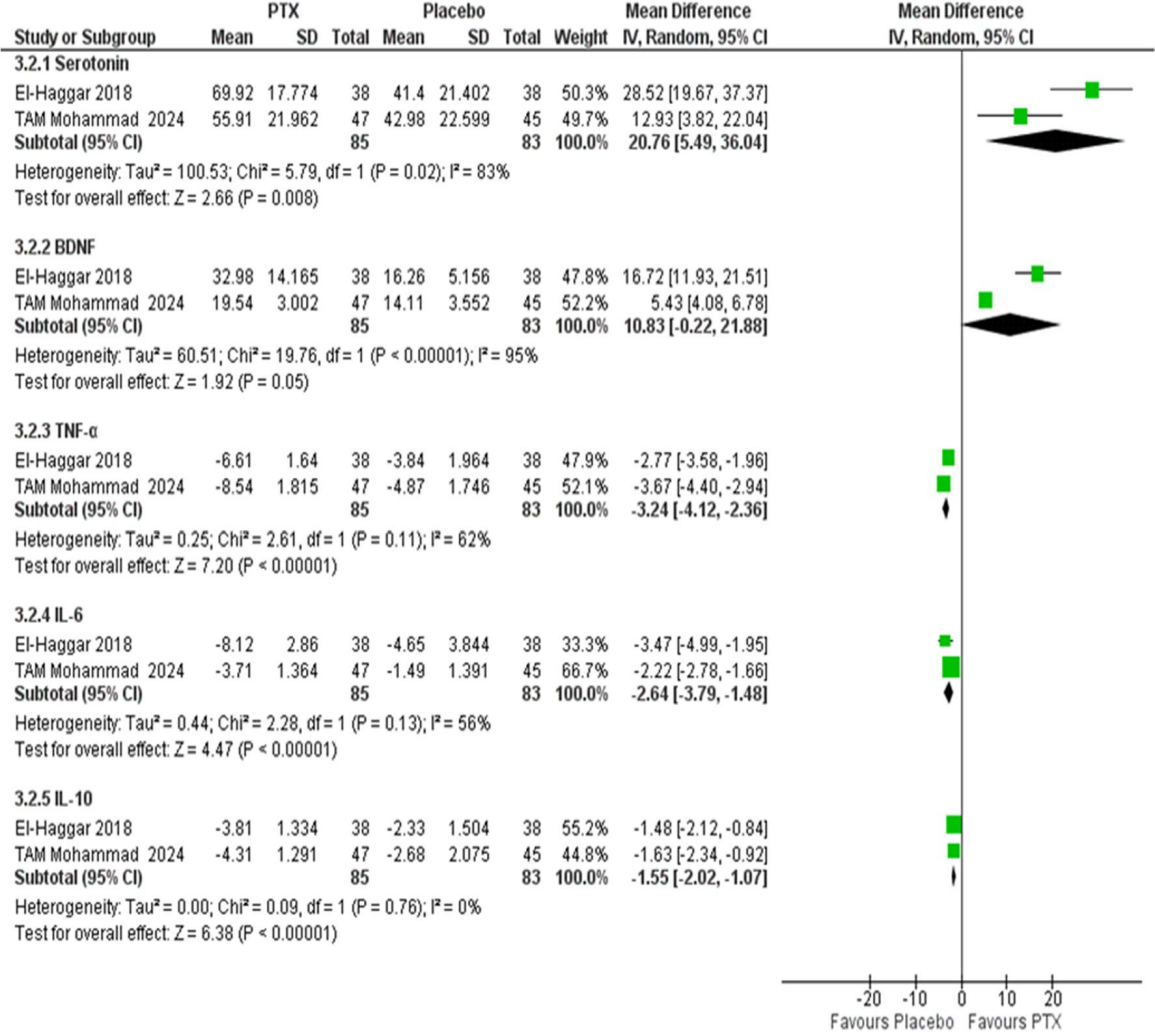
Forest plot of biological markers with a random effect model. PTX: Pentoxifylline, IV: Inverse-variance, RR: Risk ratio

Two studies reported the biological markers and the overall effect favoured PTX over placebo in all of them: Serotonin (MD = 20.76 ng/mL, 95% CI [5.49 to 36.04], *P* = 0.008), BDNF (MD = 10.83 ng/mL, 95% CI [−0.22 to 21.88], *P* = 0.05), TNF-α (MD = –3.24 pg./mL, 95% CI [–4.12 to –2.36], *P* < 0.00001), IL-6 (MD = –2.64 pg./mL, 95% CI [–3.79 to –1.48], *P* < 0.00001), and IL-10 (MD = –1.55 pg./mL, 95% CI [–2.02 to –1.07], *P* < 0.00001), as shown in the forest plot (Fig. 4).

El-Hagger et al. (2018) reported a reduction in the biological marker 8-HOdG within the PTX group compared to the placebo group (MD = −0.49, 95% CI [−0.72, −0.26]) (El‑Haggar et al. 2018).

TAM-Mohammed et al. (2024) reported a reduction in CRP within the PTX group compared to the placebo group (MD = −0.53, 95% CI [−0.89, −0.17]) and in IL-1-β within the PTX group compared to the placebo group (MD = −0.61, 95% CI [−0.87, −0.35]) (Merza Mohammad et al. 2024).

### Adverse events and side effects

**Fig. 5:**
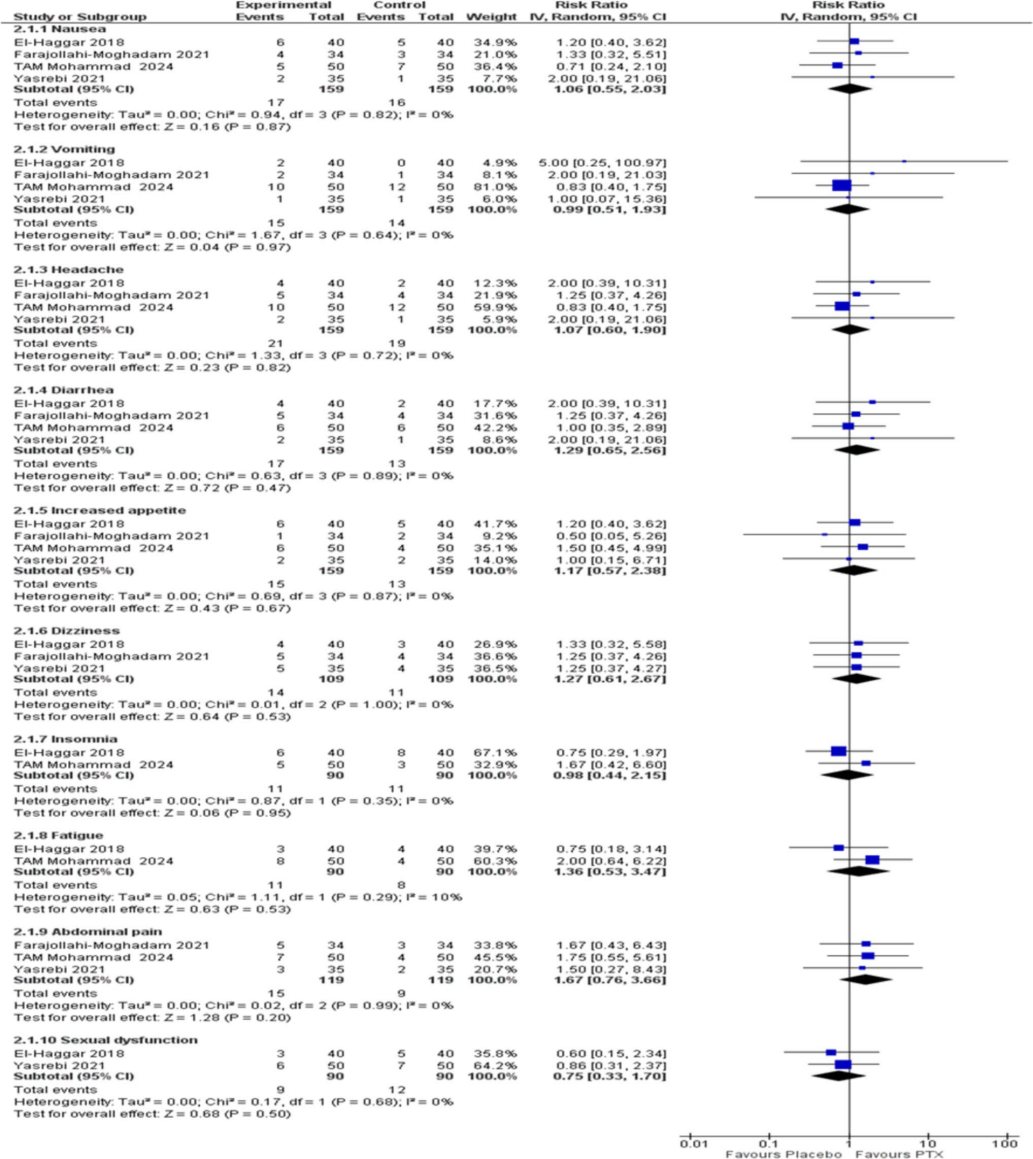
Forest plot of the reported side effects with a random effect model. PTX: Pentoxifylline, IV: Inverse-variance, RR: Risk ratio

The pooled analysis of the studies showed no statistically significant difference between PTX and placebo in all reported side effects including Nausea (RR = 1.06, 95% CI [0.55, 2.02], *P* = 0.87), Vomiting (RR = 0.99, 95% CI [0.51, 1.93], *P* = 0.97), Headache (RR = 1.07, 95% CI [0.60, 1.90], *P* = 0.82), Diarrhea (RR = 1.29, 95% CI [0.65, 2.56], *P* = 0.47), Increased appetite (RR = 1.17, 95% CI [0.57, 2.38], *P* = 0.67), Dizziness (RR = 1.27, 95% CI [0.61, 2.67], *P* = 0.53), Insomnia (RR = 0.98, 95% CI [0.44, 2.15], *P* = 0.95), Fatigue (RR = 1.36, 95% CI [0.53, 3.47], *P* = 0.53), Abdominal pain (RR = 1.67, 95% CI [0.76, 3.66], *P* = 0.20), and Sexual dysfunction (RR = 0.75, 95% CI [0.33, 1.70], *P* = 0.50) (Fig. 5).

### Discontinuation rate

**Fig. 6:**
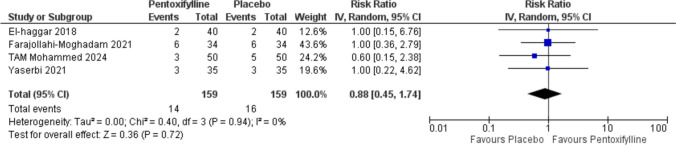
All causes of discontinuation rates. IV: Inverse-variance, CI: Confidence interval

There was no significant difference in all causes of discontinuation rate between pentoxifylline and placebo, with a risk ratio of (RR = 0.88, 95% [0.45 to 1.74], (Fig. 6).

## Discussion

Understanding mental disorders is still challenging, especially depression. The World Health Organization (WHO) has predicted that MDD will be the leading cause of disease burden by 2030 (Malhi and Mann 2018). However, approximately 30% of patients do not improve with conventional antidepressants, which primarily target the monoaminergic system (Miller et al. 2009). Our meta-analysis is the first to investigate the role of PTX, a pleiotropic drug that targets several novel pathways in depression beyond the conventional monoaminergic pathway (Cui et al. 2024).

Regarding HAM-D, PTX demonstrated a significant reduction of 3 points at 4 weeks, and an even greater reduction of 3.8 points from baseline at the posttreatment assessment, compared to the placebo group. PTX also demonstrated an increase in the response rate (≥ 50% reduction in the HAM-D score) and the remission rate (HAM-D score ≤ 7) compared to placebo. Regarding biological markers, PTX increased serotonin and BDNF and decreased IL-6, IL-10, TNF-α, and 8-OHdG compared to the placebo group. Furthermore, there were no differences between the PTX group and the placebo group regarding any of the reported side effects and all causes of discontinuation rate.

Additionally, El-Haggar et al. (2018) and TAM-Mohammed et al. (2024) both reported a statistically significant negative correlation between serum levels of serotonin and BDNF with the HAM-D score at baseline and at posttreatment. They also found a positive correlation between serum levels of TNF-α, IL-6, IL-10, and 8-OHdG with the HAM-D score before treatment and after treatment.

In the primary endpoint HAM-D score assessment, there was overall heterogeneity across the studies. To address this, a subgroup analysis was introduced, distinguishing between studies where patients received both pentoxifylline and an SSRI (El‑Haggar et al. 2018; Farajollahi-Moghadam et al. 2021; Merza Mohammad et al. 2024; Mohammad et al. 2024) and one study where patients received only pentoxifylline (Yasrebi et al. 2021). The results within the subgroup where patients received PTX and an SSRI were homogeneous.

The minimally clinical important difference (MCID) of depression is considered to be around 2–3 points based on HAM-D score (Kirsch et al. 2008). In our meta-analysis, PTX demonstrated a reduction in HAM-D score by 3 points at 4-week analysis and a greater reduction at the endpoint of −3.8 points. This suggests that PTX is an effective treatment for reducing depression symptoms.

The beneficial effects of PTX in the treatment of MDD might be through several mechanisms, among which anti-inflammatory effects are the most prominent. PTX is a phosphodiesterase inhibitor that inhibits proinflammatory cytokines such as IL-6 and TNF-α (Bah et al. 2011). MDD patients may have higher levels of cytokines such as TNF-α, CRP, and IL-6 in their blood (Lee and Giuliani 2019). Together with other cytokines, they influence the central nervous system (CNS) monoaminergic system, which is targeted by different antidepressants. Furthermore, PTX increases levels of serotonin by suppression of pro-inflammatory cytokines, which enhances serotonin bioavailability and decreases serotonin uptake by astrocytes (Capuron et al. 2017). Elevated levels of pro-inflammatory cytokines can trigger the competitive tryptophan metabolic pathways, leading to decreased serotonin, norepinephrine, and dopamine synthesis rates (Haroon et al. 2012). Additionally, inflammatory markers may impact the function of the central nervous system by activating nuclear factor-kappa B (NF-κB) and consequently inhibiting neurogenesis and affecting astrocytes by reducing the expression of glutamate transporters and elevating their release, subsequently downregulating the brain-derived neurotrophic factor (BDNF) and affecting neurogenesis (Haroon and Miller 2017; Koo et al. 2010).

Additionally, PTX is also an antioxidative agent, which reduces the levels of reactive oxygen species (ROS) that contribute to neuronal pruning and apoptosis might be implicated in MDD pathophysiology (Duman et al. 2016). Elevated cytokine levels also directly affect macrophages and neutrophils, resulting in the production of oxygen and nitrogen free radicals (Wang et al. 2015). Moreover, an included study by El-Haggar et al. (2018) reported that PTX decreased levels of 8-hydroxydeoxyguanosine (8-OHdG), a biomarker of oxidative damage (Czarny et al. 2018).

PTX increases levels of BDNF, a neuroprotective factor important in synaptic regulation and plasticity, by increasing cyclic adenosine monophosphate (cAMP) levels through PDE inhibition (Patas et al. 2014). Decreased expression of BDNF is assumed to have roles in the pathogenesis of depression [43]. Mechanistically, BDNF can exert its action by protecting against stress-induced neuronal damage and improving neurogenesis in the hippocampus, which provides a potential explanation for the observed antidepressant effect of PTX (Rosenblat et al. 2015). MDD is also associated with disruptions in cerebral blood flow, leading to hypoperfusion of some critical regions for mood regulation. Pentoxifylline is shown to improve cerebral blood flow in a dose-dependent manner in patients with cerebrovascular insufficiency (Annamaraju and Baradhi 2022; Ito et al. 1996).

In our study, we found that PTX has an antidepressant effect, which is in line with the results of Harward et al. (1979), who conducted RCT where patients with cerebral insufficiency received a daily dose of PTX and reported improvements in drive, social behavior, and mood (Harwart, 1979). Additionally, Bahlmann et al. (2006) conducted an RCT in which PTX was administered intraoperatively before cardiopulmonary bypass surgery, resulting in improvement in depression and anxiety scores postoperatively (Bahlmann et al. 2006). Furthermore, three studies were conducted on animals and reported that PTX has an antidepressant effect using forced swimming test as a measurement (Bah et al. 2011; Najjar et al. 2019; Neves et al. 2015). Moreover, a meta-analysis demonstrated that anti-inflammatory agents significantly alleviate depressive symptoms in individuals with major depressive disorder (Bai et al. 2020). Mohammad et al. (2024) disagreed with our study as they found that PTX has no antidepressant effect, which may be attributed to their inclusion of patients with treatment-resistant depression and bipolar disorder (Mohammad et al. 2024).

However, contrary to the previously proposed insights explaining how pentoxifylline exerts its function, several studies challenged these mechanisms. According to the findings of a Cochrane review, there is a lack of high-certainty evidence for the effects of PTX compared to placebo for intermittent claudication, which contradicts the proposed mechanism that PTX may help in vascular dysfunction rehabilitation (Broderick et al. 2020). Additionally, recent RCT evaluated PTX on patients with myocardial infarction and found that PTX had no significant detectable effect on endothelial dysfunction (Saeed et al. 2024). Regarding biological markers, despite the included studies found a negative correlation between HAM-D scores and BDNF and serotonin levels, there is evidence suggesting that the improvement in depression symptoms is not correlated with BDNF or serotonin levels (Moncrieff et al. 2023; Talaee et al. 2024). Therefore, while PTX may exhibit multiple mechanisms of action that align with pathways that might be implicated in MDD pathophysiology, the mechanism by which PTX may be improving depression symptoms remains unclear.

### Limitations

This is the first systematic review and meta-analysis conducted on pentoxifylline for major depressive disorder. We excluded studies involving bipolar depression and other psychiatric comorbidities to be specific.

Our study was not without limitations. The limited number of included RCTs and total sample size of just 318 patients are significant limitations. One study investigated PTX as monotherapy (Yasrebi et al. 2021), while the other three studies investigated it as an add-on to SSRIs and each study used different SSRIs (El‑Haggar et al. 2018; Farajollahi-Moghadam et al. 2021; Merza Mohammad et al. 2024). These variations could explain the heterogeneity that was found in some of our analyses. Additionally, Yasrebi et al. and Farajollahi-Moghadam et al., included patients with severe depression based on the baseline HAM-D scores, while El-haggar et al. and Mohammed et al. included patients with mild to moderate depression. Also, the mean age of patients in Yaserbi et al., was 54 years and the patients suffered from co-morbid coronary artery disease, while the patients in the other included studies were in their early 30 s. One study used 400 mg of PTX three times a day, while others used 400 mg twice daily. The geographical concentration as all studies were conducted in the Middle East. These clinical variations could limit the generalizability of our results. The studies differed in treatment duration and there was no follow-up period which prevented us from examining the sustainability of the response and long-term safety. Lastly, only two studies provided biological markers levels, which limits our ability to correlate PTX efficacy, HAM-D scores, and these biological markers through meta-regression analysis.

### Significance of work and future research

Our study provides a novel contribution to the literature by filling the gap regarding PTX efficacy and safety in major depressive disorder. The findings of our study support the role of PTX as an adjuvant therapy in MDD. These results are important in clinical practice as MDD is a challenging disease to treat which emphasizes the need for alternative therapeutic approaches considering that PTX has met or nearly exceeded the established MCID threshold for clinical relevance.

We recommend further studies to be conducted regarding the role of PTX in depression with a large sample size, longer treatment duration, and follow-up periods. Investigating PTX as monotherapy without an SSRI could provide valuable information about the exact efficacy of PTX.

## Conclusion

The study findings support the safety and efficacy of PTX as an adjuvant antidepressant agent in patients with MDD, demonstrating a significant reduction in HAM-D scores. The results of this study need to be interpreted with caution considering several limitations.

## Supplementary Information

Below is the link to the electronic supplementary material.

Supplementary file1 (DOCX 16 KB)

## Data Availability

All source data for this work (or generated in this study) are available upon reasonable request.

## References

[CR1] Annamaraju P, Baradhi KM (2022) Pentoxifylline. In: StatPearls [Internet]. Treasure Island (FL): StatPearls Publishing. Available from: http://www.ncbi.nlm.nih.gov/books/NBK559096/

[CR2] Aviado DM, Porter JM (1984) Pentoxifylline: a new drug for the treatment of intermittent claudication. Mechanism of action, pharmacokinetics, clinical efficacy and adverse effects. Pharmacotherapy 4(6):297–307. 10.1002/j.1875-9114.1984.tb03380.x 10.1002/j.1875-9114.1984.tb03380.x 6393073

[CR3] Bah TM, Kaloustian S, Rousseau G, Godbout R (2011) Pretreatment with pentoxifylline has antidepressant-like effects in a rat model of acute myocardial infarction. Behav Pharmacol 22(8):779–784. 10.1097/FBP.0b013e32834d1385 21971020 10.1097/FBP.0b013e32834d1385

[CR4] Bains N, Abdijadid S (2024) Major depressive disorder. In StatPearls. StatPearls Publishing 32644504

[CR5] Bai S, Guo W, Feng Y, Deng H, Li G, Nie H, Guo G, Yu H, Ma Y, Wang J, Chen S, Jing J, Yang J, Tang Y, Tang Z (2020) Efficacy and safety of anti-inflammatory agents for the treatment of major depressive disorder: a systematic review and meta-analysis of randomised controlled trials. J Neurol Neurosurg Psychiatry 91(1):21–32. 10.1136/jnnp-2019-320912 31658959 10.1136/jnnp-2019-320912

[CR6] Broderick C, Forster R, Abdel-Hadi M, Salhiyyah K (2020) Pentoxifylline for intermittent claudication. Cochrane Database Syst Rev 10(10):CD005262. 10.1002/14651858.CD005262.pub4 10.1002/14651858.CD005262.pub4 PMC8094235 33063850

[CR7] Capuron L, Lasselin J, Castanon N (2017) Role of adiposity-driven inflammation in depressive morbidity. Neuropsychopharmacology 42(1):115–128. 10.1038/npp.2016.123 27402495 10.1038/npp.2016.123 PMC5143483

[CR8] Chapman DP, Perry GS, Strine TW (2005) The vital link between chronic disease and depressive disorders. Prev Chronic Dis 2(1):A14 15670467 PMC1323317

[CR9] Cui L, Li S, Wang S, Wu X, Liu Y, Yu W, Wang Y, Tang Y, Xia M, Li B (2024) Major depressive disorder: hypothesis, mechanism, prevention and treatment. Signal Transduct Target Ther 9(1):30. 10.1038/s41392-024-01738-y 38331979 10.1038/s41392-024-01738-y PMC10853571

[CR10] Cumpston M, Li T, Page MJ, Chandler J, Welch VA, Higgins JP, Thomas J (2019) Updated guidance for trusted systematic reviews: a new edition of the Cochrane Handbook for Systematic Reviews of Interventions. Cochrane Database Syst Rev 10(10), ED000142. 10.1002/14651858.ED000142 10.1002/14651858.ED000142 PMC10284251 31643080

[CR11] Czarny P, Wigner P, Galecki P, Sliwinski T (2018) The interplay between inflammation, oxidative stress, DNA damage, DNA repair and mitochondrial dysfunction in depression. Prog Neuropsychopharmacol Biol Psychiatry 80(Pt C):309–321. 10.1016/j.pnpbp.2017.06.036 28669580 10.1016/j.pnpbp.2017.06.036

[CR12] Drevets WC, Thase ME, Moses-Kolko EL, Price J, Frank E, Kupfer DJ, Mathis C (2007) Serotonin-1A receptor imaging in recurrent depression: replication and literature review. Nucl Med Biol 34(7):865–877. 10.1016/j.nucmedbio.2007.06.008 17921037 10.1016/j.nucmedbio.2007.06.008 PMC2702715

[CR13] Duman RS, Aghajanian GK, Sanacora G, Krystal JH (2016) Synaptic plasticity and depression: new insights from stress and rapid-acting antidepressants. Nat Med 22(3):238–249. 10.1038/nm.4050 26937618 10.1038/nm.4050 PMC5405628

[CR14] Egger M, Smith G, Schneider M, Minder C (1997) Bias in meta-analysis detected by a simple, graphical test. BMJ (Clin Res Ed) 315(7109):629–634. 10.1136/bmj.315.7109.629 10.1136/bmj.315.7109.629 PMC2127453 9310563

[CR15] El-Haggar SM, Eissa MA, Mostafa TM, El-Attar KS, Abdallah MS (2018) The phosphodiesterase inhibitor pentoxifylline as a novel adjunct to antidepressants in major depressive disorder patients: a proof-of-concept, randomized, double-blind, placebo-controlled trial. Psychother Psychosom 87(6):331–339. 10.1159/000492619 10.1159/000492619 30205379

[CR16] Farajollahi-Moghadam M, Sanjari-Moghaddam H, GhazizadehHasemi M, Sanatian Z, Talaei A, Akhondzadeh S (2021) Efficacy and safety of pentoxifylline combination therapy in major depressive disorder: a randomized, double-blind, placebo-controlled clinical trial. Int Clin Psychopharmacol 36(3):140–146. 10.1097/YIC.0000000000000353 33724254 10.1097/YIC.0000000000000353

[CR17] Fava M, Kendler KS (2000) Major depressive disorder. Neuron 28(2):335–341. 10.1016/s0896-6273(00)00112-4 11144343 10.1016/s0896-6273(00)00112-4

[CR18] Gutiérrez-Rojas L, Porras-Segovia A, Dunne H, Andrade-González N, Cervilla JA (2020) Prevalence and correlates of major depressive disorder: a systematic review. Revista Brasileira de Psiquiatria (Sao Paulo, Brazil : 1999) 42(6):657–672. 10.1590/1516-4446-2020-0650 10.1590/1516-4446-2019-0650 PMC7678895 32756809

[CR19] Haroon E, Miller AH (2017) Inflammation effects on brain glutamate in depression: mechanistic considerations and treatment implications. Curr Top Behav Neurosci 31:173–198. 10.1007/7854_2016_40 27830574 10.1007/7854_2016_40

[CR20] Haroon E, Raison CL, Miller AH (2012) Psychoneuroimmunology meets neuropsychopharmacology: translational implications of the impact of inflammation on behavior. Neuropsychopharmacology 37(1):137–162. 10.1038/npp.2011.205 21918508 10.1038/npp.2011.205 PMC3238082

[CR21] Ito H, Kawashima R, Awata S, Ono S, Sato K, Goto R, Koyama M, Sato M, Fukuda H (1996) Hypoperfusion in the limbic system and prefrontal cortex in depression: SPECT with anatomic standardization technique. J Nucl Med 37(3):410–414 8772633

[CR22] Jacoby D, Mohler ER (2004) Drug treatment of intermittent claudication. Drugs 64(15):1657–1670. 10.2165/00003495-200464150-00004 15257627 10.2165/00003495-200464150-00004

[CR23] Jans LAW, Riedel WJ, Markus CR, Blokland A (2007) Serotonergic vulnerability and depression: assumptions, experimental evidence and implications. Mol Psychiatry 12(6):522–543. 10.1038/sj.mp.4001920 17160067 10.1038/sj.mp.4001920

[CR24] Kirsch I, Deacon BJ, Huedo-Medina TB, Scoboria A, Moore TJ, Johnson BT (2008) Initial severity and antidepressant benefits: a meta-analysis of data submitted to the Food and Drug Administration. PLoS Med 5(2):e45. 10.1371/journal.pmed.0050045 18303940 10.1371/journal.pmed.0050045 PMC2253608

[CR25] Koo JW, Russo SJ, Ferguson D, Nestler EJ, Duman RS (2010) Nuclear factor-kappaB is a critical mediator of stress-impaired neurogenesis and depressive behavior. Proc Natl Acad Sci USA 107(6):2669–2674. 10.1073/pnas.0910658107 20133768 10.1073/pnas.0910658107 PMC2823860

[CR26] Lee C-H, Giuliani F (2019) The role of inflammation in depression and fatigue. Front Immunol 10:1696. 10.3389/fimmu.2019.01696 31379879 10.3389/fimmu.2019.01696 PMC6658985

[CR27] Malhi GS, Mann JJ (2018) Depression. Lancet 392(10161):2299–2312. 10.1016/S0140-6736(18)31948-2 30396512 10.1016/S0140-6736(18)31948-2

[CR28] Merza Mohammad TA, Merza Mohammad TA, Salman DM, Jaafar HM (2024) Pentoxifylline as a novel add-on therapy for major depressive disorder in adult patients: a randomized, double-blind, placebo-controlled trial. Pharmacopsychiatry 57(4):205–214. 10.1055/a-2291-7204 10.1055/a-2291-7204 38710206

[CR29] Miller AH, Maletic V, Raison CL (2009) Inflammation and its discontents: the role of cytokines in the pathophysiology of major depression. Biol Psychiat 65(9):732–741. 10.1016/j.biopsych.2008.11.029 19150053 10.1016/j.biopsych.2008.11.029 PMC2680424

[CR30] Mohammad TAM, Mohammad TAM, Shawis TN (2024) Efficacy of pentoxifylline for the treatment of bipolar I/II patients with treatment-resistant depression: a proof-of-concept, randomized, double-blind, placebo-controlled trial. Brain Res Bull 216:111047. 10.1016/j.brainresbull.2024.111047 39128677 10.1016/j.brainresbull.2024.111047

[CR31] Moncrieff J, Cooper RE, Stockmann T, Amendola S, Hengartner MP, Horowitz MA (2023) The serotonin theory of depression: a systematic umbrella review of the evidence. Mol Psychiatry 28(8):3243–3256. 10.1038/s41380-022-01661-0 35854107 10.1038/s41380-022-01661-0 PMC10618090

[CR32] Ouzzani M, Hammady H, Fedorowicz Z, Elmagarmid A (2016) Rayyan-a web and mobile app for systematic reviews. Syst Rev 5(1):210. 10.1186/s13643-016-0384-4 27919275 10.1186/s13643-016-0384-4 PMC5139140

[CR33] Page MJ, McKenzie JE, Bossuyt PM, Boutron I, Hoffmann TC, Mulrow CD, Shamseer L, Tetzlaff JM, Akl EA, Brennan SE, Chou R, Glanville J, Grimshaw JM, Hróbjartsson A, Lalu MM, Li T, Loder EW, Mayo-Wilson E, McDonald S, … Moher D (2021) The PRISMA 2020 statement: An updated guideline for reporting systematic reviews. Int J Surg 88:105906. 10.1016/j.ijsu.2021.105906 10.1016/j.ijsu.2021.105906 33789826

[CR34] Patas K, Penninx BWJH, Bus BAA, Vogelzangs N, Molendijk ML, Elzinga BM, Bosker FJ, Oude Voshaar RC (2014) Association between serum brain-derived neurotrophic factor and plasma interleukin-6 in major depressive disorder with melancholic features. Brain Behav Immun 36:71–79. 10.1016/j.bbi.2013.10.007 24140302 10.1016/j.bbi.2013.10.007

[CR35] Rosenblat JD, McIntyre RS, Alves GS, Fountoulakis KN, Carvalho AF (2015) Beyond monoamines-novel targets for treatment-resistant depression: a comprehensive review. Curr Neuropharmacol 13(5):636–655. 10.2174/1570159x13666150630175044 26467412 10.2174/1570159X13666150630175044 PMC4761634

[CR36] Saeed A, Farouk MM, Sabri NA, Saleh MA, Ahmed MA (2024) Effect of pentoxifylline on endothelial dysfunction, oxidative stress and inflammatory markers in STEMI patients. Future Sci OA 10(1):FSO967. 10.2144/fsoa-2023-0266 10.2144/fsoa-2023-0266 PMC11137834 38817362

[CR37] Siegel AN, Rodrigues N, Nasri F, Wilkialis L, Lipsitz O, Lee Y, Gill H, Subramaniapillai M, Phan L, Majeed A, Lui LMW, Rashidian H, Ho R, Toma S, Goldstein BI, Mansur RB, McIntyre RS, Rosenblat JD (2021) Novel therapeutic targets in mood disorders: Pentoxifylline (PTX) as a candidate treatment. Prog Neuropsychopharmacol Biol Psychiatry 104:110032. 10.1016/j.pnpbp.2020.110032 32634540 10.1016/j.pnpbp.2020.110032

[CR38] Talaee N, Azadvar S, Khodadadi S, Abbasi N, Asli-Pashaki ZN, Mirabzadeh Y, Kholghi G, Akhondzadeh S, Vaseghi S (2024) Comparing the effect of fluoxetine, escitalopram, and sertraline, on the level of BDNF and depression in preclinical and clinical studies: a systematic review. Eur J Clin Pharmacol 80(7):983–1016. 10.1007/s00228-024-03680-y 38558317 10.1007/s00228-024-03680-y

[CR39] Wang W-Y, Tan M-S, Yu J-T, Tan L (2015) Role of pro-inflammatory cytokines released from microglia in Alzheimer’s disease. Ann Transl Med 3(10):136. 10.3978/j.issn.2305-5839.2015.03.49 26207229 10.3978/j.issn.2305-5839.2015.03.49 PMC4486922

[CR40] Yasrebi S-O, Momtazmanesh S, Moghaddam HS, Shahmansouri N, Mehrpooya M, Arbabi M, Ghazizadeh-Hashemi F, Akhondzadeh S (2021) Pentoxifylline for treatment of major depression after percutaneous coronary intervention or coronary artery bypass grafting: A randomized, double-blind, placebo-controlled trial. J Psychosom Res 150:110635. 10.1016/j.jpsychores.2021.110635 34627009 10.1016/j.jpsychores.2021.110635

