# Supplementary material for: Efficacy, safety and mechanistic insights of pentoxifylline in major depressive disorder: a systematic review and meta-analysis of randomized controlled trials
Source: Naunyn Schmiedebergs Arch Pharmacol. 2025 Feb 22;398(7):8125–38. doi: 10.1007/s00210-025-03845-1 (PMC12263474; doi:10.1007/s00210-025-03845-1)
Supplement: Supplementary file 1 — Supplementary file1 (DOCX 16 KB) [file 210_2025_3845_MOESM1_ESM.docx]

| **Appendix A**: databases were used to search for articles related to the following key words: | | |
| --- | --- | --- |
| **Databases** | **Search Strategy** | **Results** |
| Pubmed | (Pentoxifylline OR Oxpentifylline OR Trental OR Agapurin OR Torental OR BL-191 OR BL 191 OR Pentoxil OR Pentopak OR Lentrin OR Reotal OR Reotal OR Flexital OR Pentobid OR Pentohexal OR Agapurin OR Vasonit) AND ("depression" OR "major depressive disorder" OR "MDD" OR "clinical depression" OR "unipolar depression" OR "recurrent depression" OR "depressive disorder" OR "depressive episode" OR "treatment-resistant depression" OR "TRD" OR "chronic depression" OR "melancholic depression" OR "dysthymia" OR "persistent depressive disorder" OR "atypical depression" OR "anxiety-depression" OR "mood disorder" OR "low mood" OR "bipolar depression" OR "postpartum depression" OR "seasonal affective disorder" OR "SAD" OR "psychotic depression") | 91 |
| Scopus | TITLE-ABS-KEY ( ( "Pentoxifylline" OR "Oxpentifylline" OR "Trental" OR "Agapurin" OR "Torental" OR "BL-191" OR "BL 191" OR "Pentoxil" OR "Pentopak" OR "Lentrin" OR "Reotal" OR "Flexital" OR "Pentobid" OR "Pentohexal" OR "Vasonit" ) AND ("depression" OR "major depressive disorder" OR "MDD" OR "clinical depression" OR "unipolar depression" OR "recurrent depression" OR "depressive disorder" OR "depressive episode" OR "treatment-resistant depression" OR "TRD" OR "chronic depression" OR "melancholic depression" OR "dysthymia" OR "persistent depressive disorder" OR "atypical depression" OR "anxiety-depression" OR "mood disorder" OR "low mood" OR "bipolar depression" OR "postpartum depression" OR "seasonal affective disorder" OR "SAD" OR "psychotic depression" ) ) | 360 |
| Web of science | ((ALL=(( "Pentoxifylline" OR "Oxpentifylline" OR "Trental" OR "Agapurin" OR "Torental" OR "BL-191" OR "BL 191" OR "Pentoxil" OR "Pentopak" OR "Lentrin" OR "Reotal" OR "Flexital" OR "Pentobid" OR "Pentohexal" OR "Vasonit" ) ) AND ALL=(("depression" OR "major depressive disorder" OR "MDD" OR "clinical depression" OR "unipolar depression" OR "recurrent depression" OR "depressive disorder" OR "depressive episode" OR "treatment-resistant depression" OR "TRD" OR "chronic depression" OR "melancholic depression" OR "dysthymia" OR "persistent depressive disorder" OR "atypical depression" OR "anxiety-depression" OR "mood disorder" OR "low mood" OR "bipolar depression" OR "postpartum depression" OR "seasonal affective disorder" OR "SAD" OR "psychotic depression" )) | 71 |
| Cochrane library | ("Pentoxifylline" OR "Oxpentifylline" OR "Trental" OR "Agapurin" OR "Torental" OR "BL-191" OR "BL 191" OR "Pentoxil" OR "Pentopak" OR "Lentrin" OR "Reotal" OR "Flexital" OR "Pentobid" OR "Pentohexal" OR "Vasonit") AND ("depression" OR "major depressive disorder" OR "MDD" OR "clinical depression" OR "unipolar depression" OR "recurrent depression" OR "depressive disorder" OR "depressive episode" OR "treatment-resistant depression" OR "TRD" OR "chronic depression" OR "melancholic depression" OR "dysthymia" OR "persistent depressive disorder" OR "atypical depression" OR "anxiety-depression" OR "mood disorder" OR "low mood" OR "bipolar depression" OR "postpartum depression" OR "seasonal affective disorder" OR "SAD" OR "psychotic depression") | 44 |
| The total from the four databases: | | 565 |
| Number of duplicates: | | 124 |
| Number after removing duplication:  (By Endnote): | | 441 |
|  | |  |

**Supplementary Table 1:** Search strategies and results for each database.
